# 

**Published:** 1842-06-18

**Authors:** 


**CONTENTS OF No. 25.**

Dr. Randolph's Case of Nephritic Congestion, with enormous enlargement and Softening of the Organ; produced by violent Contusion, - - - - - - - - - - - 385
Dr. M. W. Wilson's Clinical Reports:
Case 1. Phthisis Pulmonalis, - - - - - - - 388
Case 2. Diabetes, - - - - - - - - - - 389
Case 3. Phthisis following the healing of an old Ulcer, - 389
Case 4. Chronic Gastritis and commencing Cancer of the Stomach, complicating Phthisis Pulmonalis, - - - 391
Pneumonia—Bronchitis—Laryngitis, - - - - - - 391
Endocarditis, - - - - - - - - - - - 392
ANALECTA: Description of the Inauguration of the Statue of Broussais, 393
Dr. Joseph Curtis on Aconite Plaster in Rheumatism, - - 396
M. Bouchardat's Hint for the treatment of Diabetes Mellitus, - - - - - - - - - - - 396
Mr. Bulley on Treacle (Molasses) and Water for Burns, - 397
Dr. Samuel Staniland's New Mode of Accelerating Labour, - 397
Dr. George Woollam's Case of Traumatic Tetanus, successfully treated by the Sesquicarbonate of Iron, - - - 399
Spontaneous Generation, - - - - - - - - - 400
M. Rufz on Phthisis, - - - - - - - - - 400
Digestion, - - - - - - - - - - - - 400

